# Thymic Stromal Alterations and Genetic Disorders of Immune System

**DOI:** 10.3389/fimmu.2015.00081

**Published:** 2015-02-25

**Authors:** Claudio Pignata, Roberta D’Assante, Ana E. Sousa

**Affiliations:** ^1^Department of Translational Medical Sciences, Federico II University, Naples, Italy; ^2^Instituto de Medicina Molecular, Faculdade de Medicina, Universidade de Lisboa, Lisboa, Portugal

**Keywords:** severe combined immunodeficiency, FOXN1, central tolerance, medullary thymic epithelial cells, DiGeorge syndrome, rag defects, APECED

In this specialty section of the journal, we host a topic focused on thymic stromal alterations and genetic disorders of immune system. The thymus is a specialized organ of the immune system where, through stage-specific differentiation of hematopoietic progenitor cells, fully mature and self-tolerant T cells origin. The process is strictly dependent on the link between the thymic stromal cells (TSCs), which allow the selection of a functional and self-tolerant T-cell repertoire, and the thymus tridimensional architecture. Indeed, the interaction between the developing thymocytes and the stromal cells is crucial for the development of both T cells and TSCs (1, 2). In both human and mice, the primordial thymic epithelial cells (TECs) are yet unable to fully support the T-cell development and only after the transcriptional activation of the *Forkhead-box n1* (*FOXN1*) gene, this essential function is acquired. Most of the information concerning the T-cell development came out from studies on mice carrying null mutation in *FOXN1* gene. In humans, as detailed in the Romano et al. review, the Nude/SCID phenotype is characterized by congenital alopecia of the scalp, eyebrows, and eyelashes, nail dystrophy, and a severe T-cell immunodeficiency, inherited as an autosomal recessive disorder (3). As extensively approached in the Villa et al. review, the intercellular cross-talk is also essential to support the maturation of Foxp3C natural regulatory T cells. In Omenn syndrome (OS), caused by hypomorphic Rag alterations, an infiltration of peripheral tissues by activated T cells and immune dysregulation have been found (4). The authors discuss on abnormalities of thymic microenvironment in OS with a special focus on the defective maturation of TECs, and impairment of central tolerance.

The commonest association of thymic stromal deficiency resulting in T-cell immunodeficiency is the DiGeorge syndrome (DGS), discussed in the Davies review. In this syndrome, however, the immunological impairment is highly variable, ranging from normal to a severe immune defect in rare individuals, thus suggesting that partial thymic hypoplasia may occur or that extrathymic sites of differentiation play a role in the process (5). The difference in the immunological defects between DGS and the Nude/SCID phenotypes implies that FOXN1 controlled genes are mandatory for a fully mature T-cell development process rather than the integrity of the thymus itself.

It is known that autoimmune regulator (*AIRE*) gene plays a central role in the induction of central tolerance, and different mechanisms of action have been hypothesized for this process. According to the most reliable theory, AIRE directly induces the production of tissue-specific antigens (TSA) (6). However, recent evidence suggests that another mechanism for negative selection of self-reactive thymocytes may be due to AIRE-induced differentiation of medullary TECs, and regulation of the expression of intrathymic chemokines directed to antigens presenting cells (APCs), such as thymocytes and dendritic cells (7). In their reviews, Laan and Peterson and Kisand et al. give an overview on what is known about the different mechanisms through which AIRE induces central tolerance.

The process aimed at the elimination of potential self-reactive T cells in the thymus is crucial for preventing the onset of autoimmune diseases. As discussed in the Akiyama et al. paper, medullary epithelial cells play a central role in the process through the regulation of gene expression, and, in particular, of those genes encoding for the TNF family cytokines, RANK ligand, CD40 ligand, and lymphotoxin. These genes promote the differentiation of AIRE- and TSA-expressing mTECs (8).

The mechanism by which a single *AIRE* gene can influence the transcription of such a large number of TSA within mTECs has been discussed in the Matsumoto et al. paper. Two models have been proposed. The first one implies a direct transcriptional control of *AIRE* on TSA, while the second one is based on the role of *AIRE* on the maturation program of mTECs (9).

The clinical and immunological phenotype of patients affected with autoimmune polyendocrinopathy ectodermal dystrophy (APECED), as reviewed by Petteri Arstila and Jarva, is characterized by multiple endocrine deficiencies, the most common manifestations being hypoparathyroidism, Addison’s disease, hypogonadism, and secondary amenorrhea, usually associated with the presence of autoantibodies toward the target tissues (10). However, the phenotype and, therefore, the underlying pathogenic mechanism, are even more complex, in that Chronic Mucocutaneous Candidiasis is also a prominent part of the disease. This clinical entity is related to abnormalities in the Th17-related cytokines, which are mostly involved in immune defenses against Candida (11). Finally, high titers of neutralizing autoantibodies against type I interferons, which have been shown to downregulate the expression of interferon-controlled genes, have been documented (12).

In this Research Topic, De Martino et al. focus their attention on the complexity of the APECED phenotype in that a wide variability of the clinical expression, in the presence of the same genotype alteration, has been found (13). They suggest that additional mechanisms, in addition to AIRE function, are involved in the pathogenesis of the disease. This might be helpful to understand not only the molecular basis of APECED but will also help improve diagnosis, management, and therapeutic strategies to treat this complex disease.

## Conflict of Interest Statement

The authors declare that the research was conducted in the absence of any commercial or financial relationships that could be construed as a potential conflict of interest.
